# Integrated high-fidelity preparation and analysis of photonic two-qubit states for quantum network nodes

**DOI:** 10.1038/s41598-025-29630-x

**Published:** 2025-12-18

**Authors:** Jonas C. J. Zatsch, Tim Engling, Jeldrik Huster, Louis L. Hohmann, Shreya Kumar, Stefanie Barz

**Affiliations:** 1 Institute for Functional Matter and Quantum Technologies, University of Stuttgart, 70569 Stuttgart, Germany; 2 Center for Integrated Quantum Science and Technology (IQST), University of Stuttgart, 70569 Stuttgart, Germany

**Keywords:** Optics and photonics, Physics

## Abstract

The realisation of quantum networks requires local quantum information processing at the network nodes and highly efficient transmission of quantum information across the network. Integrated photonics, based on silicon-on-insulator, is a promising platform for quantum network nodes, as it supports low-loss propagation of telecom wavelength photons, making it compatible with existing optical fibre networks. Here, we present a silicon-on-insulator integrated photonic chip, capable of bidirectional operation, enabling the preparation of arbitrary single- and two-qubit states, and performing full quantum state tomography on up to two qubits. Using our chip, we obtain preparation fidelities above ${97}{\%}$ for on-chip prepared Bell states coupled into optical fibres. Furthermore, we demonstrate that we can distribute entanglement between network nodes by preparing a two-qubit cluster state on the first node and performing full quantum state tomography on the second node, achieving a fidelity of 90.0(16)%. This result proves that our approach allows the distribution of entanglement from one chip to another. The potential of bidirectional operation makes our circuit a versatile node in telecom quantum networks, both functioning as a sender and receiver unit, a key element for the deployment of fully photonic multi-purpose quantum networks.

## Introduction

Integrated quantum photonics, based on the silicon-on-insulator (SOI) material stack, is an ideal platform for networked quantum applications. It offers a robust and scalable implementation of qubit preparation and measurement, due to its small footprint components and low-loss waveguides^1^. Additionally, SOI integrated circuits are CMOS compatible, allowing for cost-efficient and high-quality fabrication of quantum processors^1^. Since it is possible to operate SOI integrated circuits in the optical C-band, existing low-loss telecom fibre networks can be utilised as quantum channels in a quantum network^2–4^.

Quantum network nodes capable of preparing, manipulating and analysing photonic qubits are highly desirable. Implementations of quantum key distribution protocols using integrated photonics have been shown^5–11^. Furthermore, it has already been demonstrated that multi-qubit states can be prepared on a photonic chip with a high fidelity^12–15^. Additionally, the coupling of on-chip single-qubit and entangled states to an off-chip quantum channel, an optical fibre, has been realised^1,16–23^. However, an open challenge is the preparation and analysis of path-encoded photonic quantum states of more than one qubit which are coupled to a quantum channel using a single device, while still achieving high fidelities. Even more, it remains to be shown, that a maximally entangled path-encoded state can be distributed from one chip to another.

Here, we present an integrated SOI photonic circuit capable of preparing arbitrary single- and two-qubit product states, as well as maximally entangled two-qubit states encoded into two photons. Furthermore, these two-qubit states are coupled from the chip to single-mode fibres. Here, the state encoding is switched from path-encoded qubits to polarisation-encoded qubits, while maintaining the prepared state with a high fidelity of above 97%. We demonstrate its functionality by preparing three different unentangled two-qubit states and all four Bell states on-chip, coupling them to optical fibres and analysing the states using off-chip full quantum state tomography (FST) units. Furthermore, we show that the chip can also function as a receiving node in a quantum network by using it as a two-qubit FST unit and performing FST on an off-chip prepared Bell state. For all cases, we measure fidelities above 97%. Finally, we realise a proof-of-concept network consisting of two nodes by connecting two copies of our chip to each other. We prepare a maximally entangled state on the sending node, distribute it to the receiving node, where we perform FST. We measure a fidelity of 90.0(16)%, proving that our chip is capable of sharing entanglement inside the network. Our photonic chip design enables the realisation of versatile quantum network nodes, capable of acting as a sending unit of entangled quantum states, and also as a receiving unit.

## Experimental setup

**Fig. 1:**
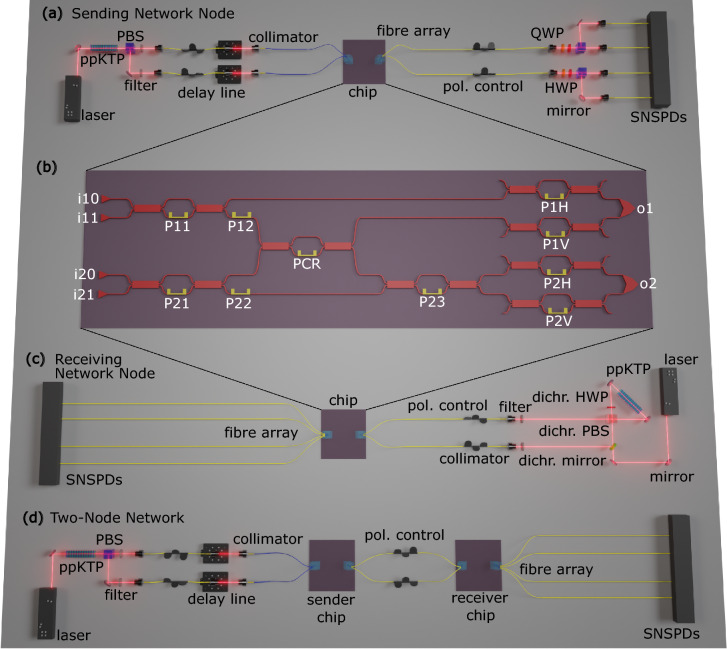
(**a**) Schematic of the setup for on-chip state preparation. Single photons are generated by a spontaneous parametric down-conversion (SPDC) source consisting of a pulsed 775nm pump laser and a periodically poled potassium titanyl phosphate crystal. A polarising beam splitter (PBS) is used to split the generated photon pairs and the photons are coupled into polarisation-maintaining fibres. The photons are coupled to the chip with the help of a fibre arrays that are aligned to the grating coupler (GCs) and two-dimensional grating coupler (2DGCs). The output state from the chip is analysed by two full quantum state tomography (FST) units consisting of a quarter-wave plate, a half-wave plate and a PBS, together with superconducting nanowire single-photon detectors (SNSPDs). (**b**) Zoom-in of the integrated photonic circuit. The integrated circuit is based on the silicon-on-insulator material platform, utilising a strip-waveguide, with a width of 450nm and a height of 220nm. On the left side of the circuit, four GCs are placed to couple light into the four qubit modes (i10, i11, i20 and i21). Four Mach-Zehnder interferometers (MZIs) consisting of, in total, six phase shifters (P11, P12, P21, P22, P23, PCR) and eight multi-mode interferometers allow for active manipulation of both qubits. The output of the circuit is connected to two 2DGCs (o1 and o2) on the right side. These 2DGCs transform the path-encoded qubits to two polarisation-encoded qubits and emit them out of the chip plane. In each of the arms of the 2DGCs a MZI (P1H, P1V, P2H, P2V) is placed for active compensation of imbalances in the 2DGCs. The total chip size, including area for wirebonding, is ${4}\textrm{mm}\, \times \,{4}\textrm{mm}$. (**c**) Schematic of the Bell state source used in the on-chip tomography experiment. Here, the crystal is placed within a Sagnac-type interferometer and the pump beam is diagonally polarised to pump the crystal from both directions^24^. In this configuration, the photons generated from both directions of pumping are indistinguishable, resulting in the generation of polarisation entangled photon pairs. The photons propagating along the pump beam are separated using a dichroic mirror and coupled into single-mode fibres. Additionally, 1.5nm band-pass filters centered at 1550nm are used to ensure spectral indistinguishability. We connect each of the two photons of the source to one of the 2DGCs. An SNSPD channel is connected to each of the four GCs i10, i11, i20 and i21. (**d**) Schematic of the setup for the chip-to-chip experiment. We place two copies of our chip in two individual coupling setups and connect them using single-mode fibres. Again, we use a linear SPDC source to generate a pair of single photons. Four SNSPDs are utilised to detect a photon in each qubit mode.

Our SOI-based photonic circuit features four waveguides acting as spatial modes. To encode our qubits we use dual-rail encoding, thus our chip encodes two qubits. Here, a photon in the upper waveguide corresponds to $|0\rangle$, and a photon in the lower waveguide to $|1\rangle$. The integrated circuit features Mach-Zehnder interferometers (MZIs) consisting of thermo-optic phase shifters and multi-mode interferometers (MMIs), allowing for active control of the chip to prepare arbitrary two-qubit states (see Fig. 1). The phase shifters are characterized by applying an increasing current and measuring the signal at the MZI output, allowing us to determine a relationship between the applied current and the set phase. Thermal cross-talk is not included in this characterisation, as the phase shifters are relatively far apart from eachother and we did not observe an immediate effect. A key element for converting path-encoded qubits to polarisation-encoded qubits, whilst conserving the qubit state, are two-dimensional grating couplers (2DGCs), placed on the right side of the circuit^25^. For this, light coming from the upper waveguide is emitted out of the chip plane horizontally polarised, and light coming from the lower waveguide is emitted vertically polarised, and vice versa when going from polarisation- to path-encoding, implementing the transformation

1 $$\begin{aligned} \alpha |0\rangle + \beta |1\rangle \leftrightarrow \alpha |H\rangle + \beta |V\rangle \text {,} \end{aligned}$$

with $\alpha$ and $\beta$ being complex amplitudes (see Methods section for a more detailed analysis of the 2DGCs’ behaviour).

Pairs of single photons are generated in a SPDC source, based on periodically poled potassium titanyl phosphate (ppKTP) crystals (see Fig. 1 for details). The single photons are coupled into fibres and into the photonic chip. To analyse the state prepared on our chip, the output state is again collected by two fibers and analysed by two FST units, which are connected to superconducting nanowire single-photon detectors (SNSPDs). We exchange the linear photon source and the tomography stages for a Sagnac-type Bell state source to demonstrate the chip’s capability to act as a two-qubit tomography unit. In a third experiment, to fully demonstrate our chip’s potential as a quantum network node, we connect two copies of our chip using single-mode fibres. One chip, acting as a sending node, is connected to the linear SPDC source from our first experiment. The second chip, acting as a receiving node, is connected to four SNSPDs. The photonic chip and the setup are presented in Fig. 1.

## Results

As a first test of our experimental setup, we perform a Hong–Ou–Mandel (HOM) experiment at the MZI containing the phase shifter PCR by setting the splitting ratio to 50%^26^.

**Fig. 2:**
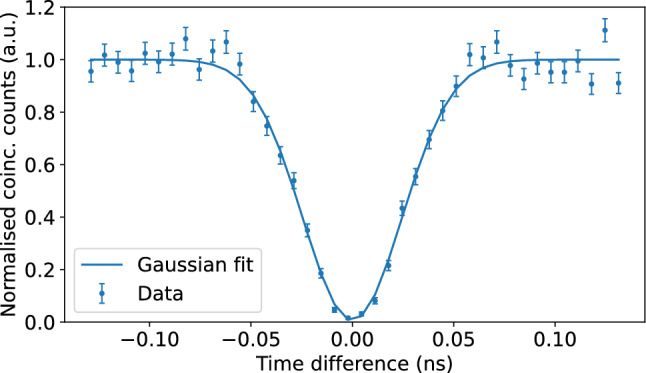
Hong–Ou–Mandel (HOM) interference measured at the crossing Mach-Zehnder interferometer (MZI) (containing PCR) using the two photons emitted by the linear spontaneous parametric down-conversion source shown in Fig. 1. Error bars are estimated from Poissonian photon statistics. The temporal shift in arrival time is introduced by changing the path length to the MZI of one of the two photons using the off-chip delay line. We measure a HOM visibility of 99.5(4)%.

From the measured data (see Fig. 2), we obtain a visibility of 99.5(4)%, demonstrating the high quality of our photon source and the chip operation.

To demonstrate the functionality of the chip for state generation, we prepare three unentangled two-qubit states. By setting $\phi _\text {PCR}$ and $\phi _\text {P23}$ accordingly (see Table 1), we completely separate the two on-chip qubits from each other, leaving an MZI plus an outer phase shifter in each qubit. When sending in a photon in the upper input, such an MZI implements the transformation

2 $$\begin{aligned} |0\rangle _k \rightarrow \alpha _k |0\rangle _k + \beta _k |1\rangle _k \end{aligned}$$

with $k\in \{1,2\}$ labelling the qubit and $\alpha _k$ and $\beta _k$ being dependent on the phase settings as listed in Table 1. We choose to prepare the eigenstates of the Pauli matrices $|H,V\rangle$, $|+,-\rangle$, and $|+_\text {i},-_\text {i}\rangle$ (see Table 1 for detailed settings). The states are then coupled out of the chip by the 2DGCs and we perform FST using the off-chip tomography stages (see Fig. 1).

**Fig. 3:**
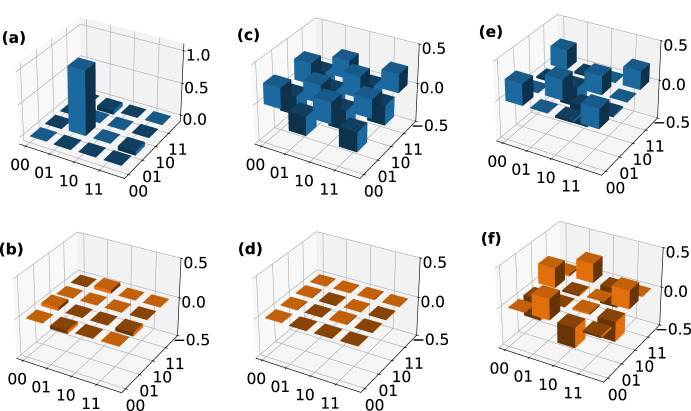
Real and imaginary parts of the reconstructed density matrices of (**a, b**) $|H,V\rangle$, (**c, d**) $|+,-\rangle$, and (**e, f**) $|+_\text {i},-_\text {i}\rangle$. The states are prepared on chip by setting the phases according to Table 1 and coupled to a pair of optical fibres via the two-dimensional grating couplers. Each photon is send to a full quantum state tomography (FST) unit as shown in Fig. 1. We perform FST and recover the density matrix of each state by a maximum likelihood estimation on the measured coincidence counts. The corresponding fidelities and purities of each state are listed in Table 1.

We perform maximum likelihood estimation (MLE) to analyse the results. For the recovered density matrix $\rho _\text {exp}$, we calculate the fidelity *F* using the equation

3 $$\begin{aligned} F = \left( \text {tr} \sqrt{\sqrt{\rho _\text {target}} \rho _\text {exp} \sqrt{\rho _\text {target}}}\right) ^2 \end{aligned}$$

and the purity using

4 $$\begin{aligned} P = \text {tr}\left( \rho _\text {exp}^2 \right) \end{aligned}$$

with $\rho _\text {target}$ being the theoretically expected density matrix (Fig. 3). This leads to an average fidelity of 99.319(10)% and an average purity of 99.41(2)%. The reconstructed density matrices of the three states are visualised in Fig. 3.

Following this, we prepare the set of all four Bell states on-chip. Choosing the phase settings according to Table 1 and post-selecting on one photon in each qubit mode, we prepare

5 $$\begin{aligned} \begin{aligned} |00\rangle&\rightarrow \text {cos}\left( \frac{\phi _\text {P23}}{2}\right) \left( |00\rangle -\text {e}^{\text {i}\phi _\text {P12}}|11\rangle \right) \\&+ \text {sin}\left( \frac{\phi _\text {P23}}{2}\right) \left( |01\rangle +\text {e}^{\text {i}\phi _\text {P12}}|10\rangle \right) \,\text {,} \end{aligned} \end{aligned}$$

up to a global phase. This procedure of preparing Bell states using linear optics has a success probability of 50%. Again, we couple the on-chip prepared states out of the chip using the 2DGCs and perform off-chip FST.

**Fig. 4:**
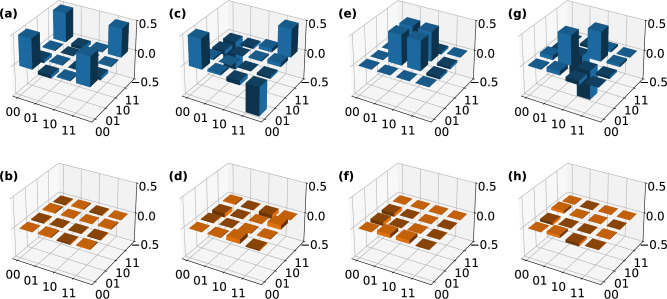
Real and imaginary part of the recovered density matrices of (**a, b**) $|\Phi ^\text {+}\rangle$, (**c, d**) $|\Phi ^\text {-}\rangle$, (**e, f**) $|\Psi ^\text {+}\rangle$, and (**g, h**) $|\Psi ^\text {-}\rangle$. The state is prepared on the chip by setting the phases according to Table 1 and coupled to a pair of optical fibres via the two-dimensional grating couplers. Each photon is send to a full quantum state tomography (FST) unit as shown in Fig. 1. We perform FST and recover the density matrix of each state by a maximum likelihood estimation on the measured coincidence counts. The corresponding fidelities and purities of each state are listed in Table 1.

The measured states’ density matrices are presented in Fig. 4 and we determine an average fidelity of 97.69(3)%, and an average purity of 97.93(7)%. The error is estimated from Poissonian distributed coincidence counts. To our knowledge, these fidelities are among the highest reported for such an experiment.

**Table 1:** Phase settings for the on-chip preparation of all six eigenstates of the three Pauli operators and all four Bell states.

State/basis	$\phi _\text {P11}$	$\phi _\text {P12}$	$\phi _\text {P21}$	$\phi _\text {P22}$	$\phi _\text {PCR}$	$\phi _\text {P23}$	Fidelity (%)	Purity (%)
$|H,V\rangle$	$\pi$	0	0	0	$\pi$	$\pi$	99.274(15)	99.98(3)
$|+,-\rangle$	$\pi /2$	$\pi$	$\pi /2$	0	$\pi$	$\pi$	99.553(14)	99.40(3)
$|+_\text {i},-_\text {i}\rangle$	$\pi /2$	$3\pi /2$	$\pi /2$	$\pi /2$	$\pi$	$\pi$	99.13(2)	98.84(5)
$|\Phi ^+\rangle$	$\pi /2$	$\pi$	$\pi /2$	0	0	0	98.13(7)	97.70(14)
$|\Phi ^-\rangle$	$\pi /2$	0	$\pi /2$	0	0	0	97.00(7)	98.09(15)
$|\Psi ^+\rangle$	$\pi /2$	0	$\pi /2$	0	0	$\pi$	98.25(6)	98.36(13)
$|\Psi ^-\rangle$	$\pi /2$	$\pi$	$\pi /2$	0	0	$\pi$	97.39(7)	97.57(15)
$|C\rangle$	$\pi /2$	0	$\pi /2$	0	0	$\pi /2$	90.0(16)	91(3)
*XX*	$\pi /2$	0	$\pi /2$	0	$\pi$	$\pi$	-	-
*YY*	$\pi /2$	$\pi /2$	$\pi /2$	$\pi /2$	$\pi$	$\pi$	-	-
*ZZ*	$\pi$	0	$\pi$	0	$\pi$	$\pi$	-	-

The phase shifters P1H, P1V, P2H, and P2V are set to compensate for imbalances introduced by the two-dimensional grating couplers (2DGCs) during the experiments. The chip layout and phase shifter locations are shown in Fig. 1.

The states prepared on-chip are coupled out of the chip using 2DGCs and analysed by two off-chip full quantum state tomography units. From the measured coincidence counts, the density matrix of each state is recovered using maximum likelihood estimation. Here, the determined fidelity (see Eq. 3) and purity (see Eq. 4) of each state are given. Additionally, the phase settings to prepare a two-qubit cluster state $|C\rangle$, which is sent from the sender chip to the receiver chip, and the determined fidelity and purity are given.

We also list the phase settings for setting the on-chip bases *X*, *Y*, and *Z*.

**Fig. 5:**
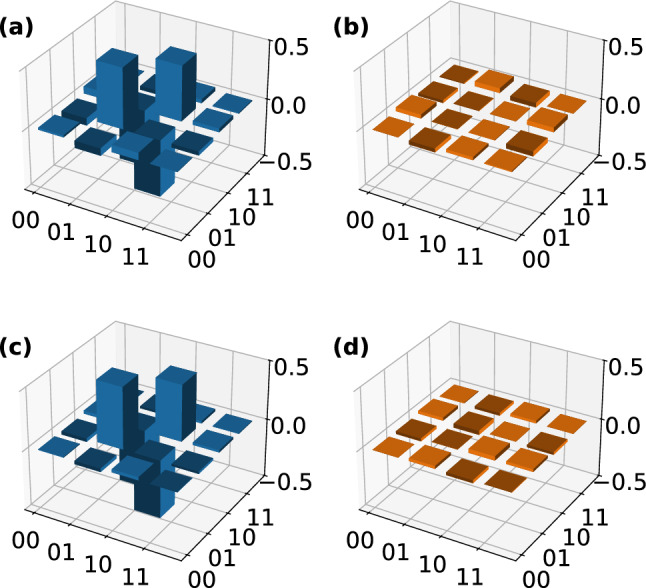
We prepare the state $|\Psi ^-\rangle$ off-chip and couple it into the photonic chip, with the chip functioning as two full quantum state tomography (FST) units. We perform FST on the state and analyse the results by maximum likelihood estimation. Here, the (**a**) real and (**b**) imaginary parts of the recovered density matrix are visualised. The fidelity of the measured state is 97.3(2)% and its purity is 97.6(5)%. As a benchmark for the on-chip FST units, we also perform off-chip FST on the prepared Bell state. The recovered density matrix’ (**c**) real and (**d**) imaginary parts are visualised. We determine a fidelity of 99.109(6)% and a purity of 99.321(15)%.

The chip’s functionality is not limited to preparing any arbitrary one- or two-qubit state, but it is also capable of measuring any one- or two-qubit state (Fig. 5). To demonstrate this, we utilise the chip as a two-qubit FST unit for two-qubit states that are prepared off-chip in polarisation-encoding and then sent to the chip, where the encoding is changed to path-encoding. For that, we utilise an SPDC source in a Sagnac configuration, which generates the state $|\Psi ^-\rangle$ (see Fig. 1). By adjusting the phase shifters according to Table 1, we can project each qubit onto one of the three bases *X*, *Y*, and *Z*. The density matrix of the measured state, is visualised in Fig. 5 and exhibits a fidelity of 97.3(2)% and a purity of 97.6(5)%. We benchmark our result by an off-chip FST of the same state, yielding a fidelity of 99.109(6)% and a purity of 99.321(15)%. This is in good agreement with the on-chip measurement with a relative fidelity

6 $$\begin{aligned} F_\text {rel} = \frac{F_\text {on-chip}}{F_\text {off-chip}} = 98.2(2){\%} \text {,} \end{aligned}$$

demonstrating the functionality of our chip as a receiving unit in a quantum network. The difference in fidelity between the measurements is likely caused by non-unitary cross-talk in the 2DGC, which reduces the fidelity of the state in an irreversible way.

**Fig. 6:**
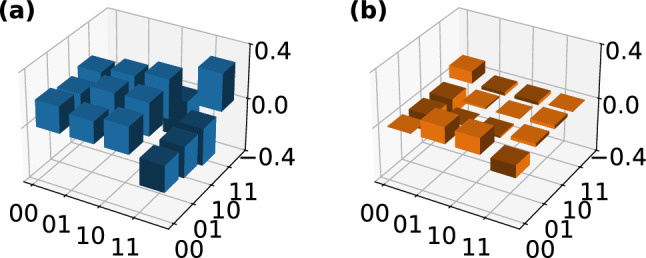
Using the sender chip we prepare a two-qubit cluster state $|C\rangle$ and send it to the receiver chip. Using the receiver chip, we perform on-chip full quantum state tomography on the state and analyse the results by maximum likelihood estimation. Here, the (**a**) real and (**b**) imaginary parts of the recovered density matrix are visualsed. The fidelity of the measured state is 90.0(16)% and its purity is 91(3)%.

Having shown the potential of our circuit to act as a sending and as a receiving unit, we demonstrate its application as a flexible quantum network node by connecting two copies of our chip (see Fig. 1). The first chip acts as a sending unit by preparing the two-qubit cluster state

7 $$\begin{aligned} |C\rangle = \frac{1}{2} \left( |00\rangle + |10\rangle + |01\rangle - |11\rangle \right) \end{aligned}$$

on chip. The output photons are connected to a second copy of the chip, which acts as a receiving unit and performs measurements in combinations of *X*, *Y* and *Z* bases. We perform on-chip FST using the receiver chip and use MLE to analyse the results (Fig. 6). All phase settings are given in Table 1. The recovered density matrix is visualised in Fig. 6. We determine a fidelity of 90.0(16)% and a purity of 91(3)%, which confirms the successful transmission of a maximally entangled state from one chip to another.

## Conclusion

In this work, we present a photonic chip that is capable of preparing arbitrary one- or two-qubit states and can act as a measurement unit for up to two qubits. We demonstrate its state preparation functionality by preparing three different unentangled two-qubit states and the set of all four Bell states. For the unentangled states, we measure an average fidelity of 99.319(10)%, while the average fidelity for the Bell states is measured to be 97.69(3)%. Furthermore, we use the chip to perform FST on an off-chip prepared Bell state and measure a fidelity of 97.3(2)%. We combine these two operational modes of our chip by connecting two copies of it, realising a proof-of-concept quantum network. We prepare a maximally entangled state on the first chip and perform FST on the second, achieving a fidelity of 90.0(16)% and therefore proving that our chip is capable of sharing entanglement across the network.

The demonstrated combined functionality of state preparation and detection with high fidelities makes our chip a versatile node in a quantum network. Expanding the circuit with additional GCs and MZIs would allow for dynamic switching between both configurations on-chip during operation. Due to our chip being low in power consumption and able to prepare Bell states, it presents a step towards the realization of an integrated sender unit in entanglement-based quantum key distribution protocols using satellites. In addition, our approach allows for scaling to a higher number of qubits by increasing the number of components in the state preparation circuit. Future steps include the cointegration of on-chip photon sources and detectors with our chip, enabling a fully integrated operation of the quantum network node. Furthermore, for applications requiring high switching-speeds, the integration of our chip layout on different material platforms allowing for electro-optic switching is of interest^27^.

## Methods

### Imperfections of 2DGCs

**Fig. 7:**
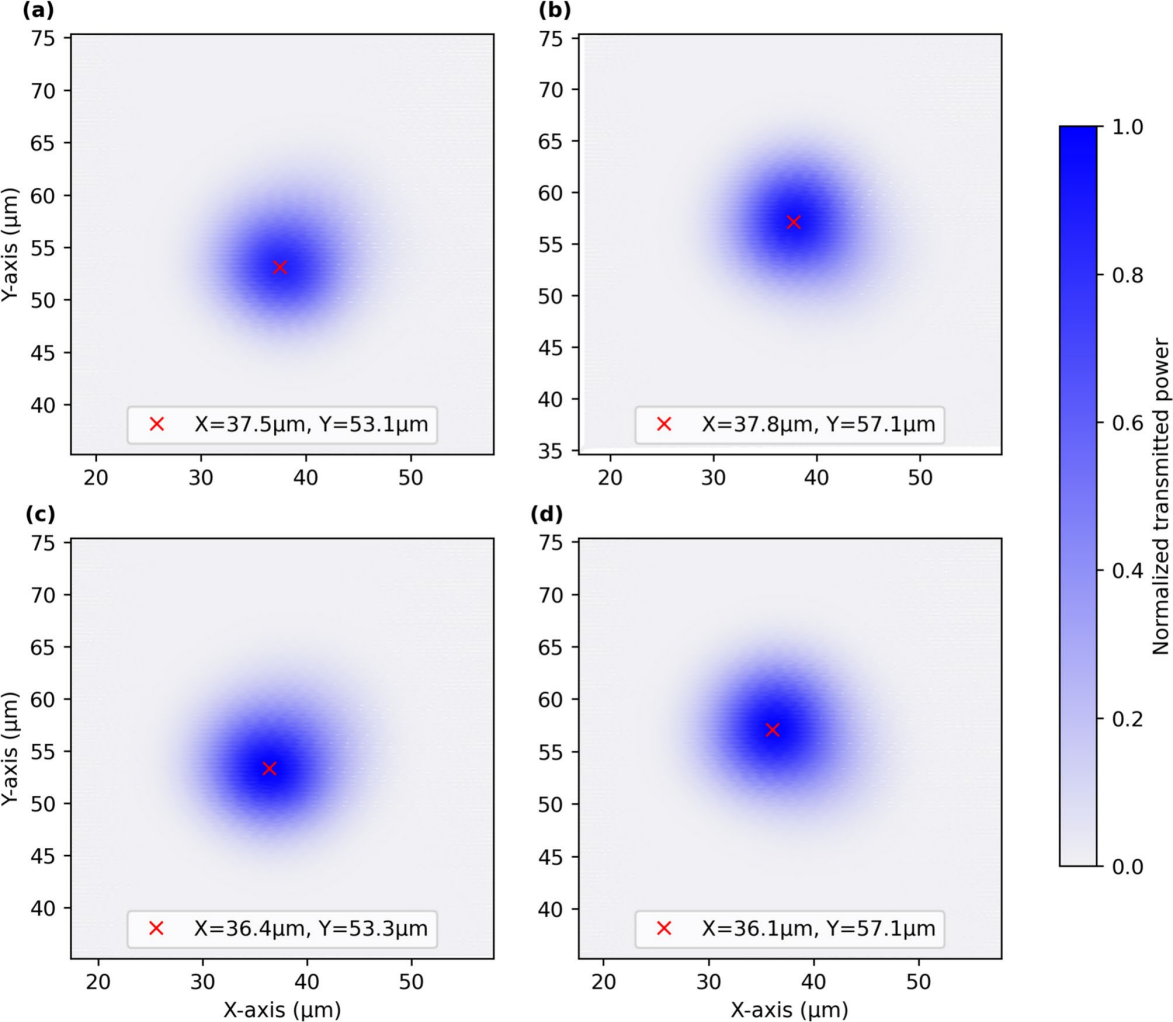
Area scans of the emitted spatial light mode of both two-dimensional grating couplers (2DGCs). A classical 1550nm light source is coupled into the (**a**) upper and (**b**) lower arm of the first 2DGC and into the (**c**) upper and (**d**) lower arm of the second 2DGC. The red cross indicates the intensity maximum.

The two 2DGCs transform the photon’s state from the path-encoding to the polarisation-encoding, they thus implement the transformation

8 $$\begin{aligned} \begin{aligned} |0\rangle&\leftrightarrow |H\rangle \\ |1\rangle&\leftrightarrow |V\rangle \end{aligned} \end{aligned}$$

with $|0\rangle$ and $|1\rangle$ referring to path-encoded states and $|H\rangle$ and $|V\rangle$ to polarisation-encoded states. Furthermore, for an ideal 2DGC, we expect both polarisations to be emitted from the coupler into the same spatial position, where we place a fibre to couple in the photons. Our experimental characterisation shows a small deviation from the ideal behaviour. From our data, we estimate a cross-talk of about 1%, corresponding to a path-encoded state being converted into the wrong polarisation. In addition, the two spatial modes corresponding to the two polarisation states slightly deviate from eachother (see Fig. 7). We compensate for this imbalance in coupling efficiency by introducing additional MZIs.

### Transmission efficiency

We estimate the end-to-end transmission efficiency by routing classical laser light through our circuit into one of the two arms of a 2DGC and measure the intensity at the output. From that, we extract the transmission efficiencies $\tau$ to be

9 $$\begin{aligned} \begin{aligned} \tau _0&= 18.8(4){\%} \\ \tau _1&= 18.4(4){\%} \text {,} \end{aligned} \end{aligned}$$

with the index referring to the 2DGC ports.

From characterisation measurements of waveguide loss test structures, we estimate the waveguide loss to be -3.4(4)dB/cm. Including this in the analysis of the transmission efficiencies, we can determine the coupling efficiencies of the couplers to be

10 $$\begin{aligned} \begin{aligned} \epsilon _\text {1D}&= 60.3(5){\%} \\ \epsilon _\text {2D,0}&= 32.6(17){\%} \\ \epsilon _\text {2D,1}&= 31.9(16){\%} \text {.} \end{aligned} \end{aligned}$$

## Data Availability

Data used for the study are available from the authors on reasonable request.
